# Differential Expression of Genes Involved in Host Recognition, Attachment, and Degradation in the Mycoparasite *Tolypocladium ophioglossoides*

**DOI:** 10.1534/g3.116.027045

**Published:** 2016-01-20

**Authors:** C. Alisha Quandt, Yanming Di, Justin Elser, Pankaj Jaiswal, Joseph W. Spatafora

**Affiliations:** * Department of Botany and Plant Pathology, Oregon State University, Corvallis, Oregon, 97331; † Department of Statistics, Oregon State University, Corvallis, Oregon, 97331

**Keywords:** PTH11 GPCR, chitinase, adhesin, RNA-Seq, *Elaphomyces*

## Abstract

The ability of a fungus to infect novel hosts is dependent on changes in gene content, expression, or regulation. Examining gene expression under simulated host conditions can explore which genes may contribute to host jumping. Insect pathogenesis is the inferred ancestral character state for species of *Tolypocladium*, however several species are parasites of truffles, including *Tolypocladium ophioglossoides*. To identify potentially crucial genes in this interkingdom host switch, *T. ophioglossoides* was grown on four media conditions: media containing the inner and outer portions of its natural host (truffles of *Elaphomyces*), cuticles from an ancestral host (beetle), and a rich medium (Yeast Malt). Through high-throughput RNASeq of mRNA from these conditions, many differentially expressed genes were identified in the experiment. These included PTH11-related G-protein-coupled receptors (GPCRs) hypothesized to be involved in host recognition, and also found to be upregulated in insect pathogens. A divergent chitinase with a signal peptide was also found to be highly upregulated on media containing truffle tissue, suggesting an exogenous degradative activity in the presence of the truffle host. The adhesin gene, *Mad1*, was highly expressed on truffle media as well. A BiNGO analysis of overrepresented GO terms from genes expressed during each growth condition found that genes involved in redox reactions and transmembrane transport were the most overrepresented during *T. ophioglossoides* growth on truffle media, suggesting their importance in growth on fungal tissue as compared to other hosts and environments. Genes involved in secondary metabolism were most highly expressed during growth on insect tissue, suggesting that their products may not be necessary during parasitism of *Elaphomyces*. This study provides clues into understanding genetic mechanisms underlying the transition from insect to truffle parasitism.

Fungal pathogens and parasites are common elements of most ecosystems, have evolved numerous times from different ancestral ecologies, and often involve jumps between disparate hosts. *Tolypocladium* is a genus in Ophiocordycipitaceae (Hypocreales, Sordariomycetes), which is a large family of fungi that are primarily pathogens of insects (Sung *et al.* 2007; Quandt *et al.* 2014). While there are a few species of insect pathogenic *Tolypocladium*, most species in the genus are parasites of truffle fruiting bodies (Quandt *et al.* 2014). The truffle parasitizing members of *Tolypocladium* have a narrow host range restricted to the ectomycorrhizal genus *Elaphomyces* (Eurotiales, Ascomycota). Despite having very different ecologies, the insect pathogenic and mycoparasitic species of *Tolypocladium* shared a recent common ancestor from approximately 50–70 million years ago and appear to be the product of a rapid radiation event (Sung *et al.* 2007, 2008; Spatafora *et al.* 2007; Quandt *et al.* 2014). *Tolypocladium*, therefore, represents an ideal system for investigating the mechanisms associated with host-jumping between distantly related hosts. The genome of *Tolypocladium ophioglossoides*, an *Elaphomyces* parasite, was recently sequenced (Quandt *et al.* 2015a) and found to be similar in genome size (31.2 Mb) and gene number (10,134) to its insect pathogenic relatives, including the congener and beetle pathogen *T. inflatum* (Bushley *et al.* 2013) and the model insect pathogen *Metarhizium robertsii* (Gao *et al.* 2011).

Another larger group of mycoparasites within Hypocreales is the family Hypocreaceae (Kubicek and Harman 1998; Rossman *et al.* 1999), which includes industrially important cellulose-producing species of *Trichoderma* (Samuels 1996; Harman and Kubicek 1998). Several *Trichoderma* spp. are used in the biocontrol of plant-pathogenic fungi (Evans *et al.* 2003; Schuster and Schmoll 2010) and studies have examined which genes are expressed during growth on plant-pathogenic fungi. Seidl *et al.* (2009), for example, used expressed sequence tags (ESTs) from a custom *Trichoderma atroviride* library and found mycoparasitism-induced upregulation of genes involved in posttranslational processing, amino acid metabolism, and catabolism of lipids and amino sugars. Reithner *et al.* (2011) found differential expression of several proteases when cultures of *Tr. atroviride* were grown antagonizing the plant pathogen *Rhizoctonia solani*. Another study used reverse transcription PCR (along with densitometry) and chitinase-specific primers to discover upregulation of chitinase gene family members during mycoparasitism in *Tr. virens* (see below, Gruber *et al.* 2011). None of the studies involved transcriptome analysis of the genome wide expression of *Trichoderma* spp. when grown on fungal hosts.

A crucial step in a parasite’s life history involves the ability to recognize its specific host and differentiate it from other objects it encounters. G-protein-coupled receptors (GPCRs) are seven transmembrane domain proteins embedded in the plasma membrane of eukaryotic cells that play critical roles in sensing the environment and initiating the signal transduction of downstream regulatory pathways (Rosenbaum *et al.* 2009). In fungi, one type of GPCR gene, called PTH11-related, is abundant in pathogenic species of the class Sordariomycetes (Kulkarni *et al.* 2005; Zheng *et al.* 2010). Expression of these GPCRs has been examined in insect pathogenic relatives of *Tolypocladium*. In the insect pathogen *M. acridum* (Clavicipitaceae, Hypocreales), which specializes in only parasitizing locusts and grasshoppers (Acrididae), different PTH11-related receptor paralogs were found to be upregulated under host and nonhost conditions, while *M. roberstii*, a generalist pathogen of insects, used a single PTH11-related GPCR (MAA_06258) to recognize both insect hosts (Gao *et al.* 2011). It is currently unknown if GPCRs function in a similar way in mycoparasites and the recognition of their hosts.

Attachment to host tissue is also an important process in pathogenicity. In EST studies of *M. robertsii* grown on insect hemolymph and plant root exudate (Wang *et al.* 2005), two adhesins, MAD1 and MAD2, were identified and highly expressed. A subsequent study demonstrated that conidia of *Mad1*knockout mutants were unable to adhere to locust wings, whereas similar *Mad2* knockout mutants were unable to attach to onion epidermis, and that *Mad1* deletion also interfered with conidial germination and hyphal differentiation (Wang and St Leger 2007). These findings suggest that MAD1 is important for attachment to insects in *M. robertsii*, while MAD2 is necessary for interactions with plant tissues and the rhizosphere. Studies in other fungal pathogens including *Candida* spp. and *Coccidioides immitis* have also demonstrated the importance of adhesins in pathogenicity (Cormack *et al.* 1999; Hung *et al.* 2002; Argimón *et al.* 2007).

The correlation between the quantity of certain carbohydrate active enzymes (CAZymes) (Henrissat and Daviest 1997) and life history has been well demonstrated in fungi (Martin *et al.* 2008; Sharpton *et al.* 2009; Floudas *et al.* 2012). Further understanding of the quantitative differences of lineage specific losses/expansions and incomplete lineage sorting of CAZymes can provide more information about the utilization of novel host resources. The number of chitinases, specifically those in CAZyme family GH18, is expanded in the sequenced genomes of the mycoparasitic lineage Hypocreaceae (Kubicek *et al.* 2011). In *Tr. virens*, differential expression of chitinases was specifically examined during various environmental and nutritional conditions as well as in confrontation assays with fungal hosts (Gruber *et al.* 2011). A subset of the fungal/bacterial Class V chitinases in the subgroup C were found to be upregulated during mycoparasitic conditions and in media containing cell walls of fungal hosts (Gruber *et al.* 2011).

In addition to using a variety of nutritional sources, hypocrealean species are also prolific producers of secondary metabolites. Some of these metabolites are thought to play an important role in pathogenesis. For example, cyclic peptides called destruxins are produced by species of *Metarhizium* and are known to have insecticidal properties. Also, peptaibiotics, produced by members of Hypocreaceae (Whitmore and Wallace 2004), form ion channels and have antifungal and antibiotic activity by inserting into plasma membranes leading to disruption of membrane potential (Chugh and Wallace 2001). One prevailing hypothesis proposes that the numerous secondary metabolites produced by *Trichoderma* spp. play a role in the mycoparasitism process (Schirmbock *et al.* 1994; Röhrich *et al.* 2012). However, studies examining the initial stages of mycoparasitism in *Tr. atroviride* and *Tr. harzianum* revealed that genes involved in secondary metabolism were down-regulated (Vinale *et al.* 2009; Reithner *et al.* 2011).

In order to understand the evolution of mycoparasitism in *Tolypocladium*, it is key to understand the complete set of genes that are differentially regulated when parasites first encounter their hosts. To this aim, RNA was sampled from the sequenced strain of *T. ophioglossoides* that was grown under four different growth conditions: minimal media amended with the tough exterior peridium (EMP) or powdery inner spore mass called a gleba (EMG) of its *Elaphomyces* host, and media containing insect cuticle (CUT) to represent the ancestral host affiliation for the genus, as well as a standard “rich” Yeast Malt medium (YM). Analysis of differential gene expression was used to determine which genes may play an important role in *Elaphomyces* recognition, with relevant comparisons to related insect pathogens and mycoparasites.

## Materials and Methods

### Media preparation

Yeast Malt (YM) broth was prepared by dissolving 4 g of yeast extract and 20 g of malt extract in 1 L of deionized water. Minimal media (MM) was made following the recipe in St Leger *et al.* (1999). Truffles of *Elaphomyces muricatus* were cleaned of debris and surface sterilized using 85% ethanol for 30 sec. A spatula was used to physically remove the warty outer peridium; the truffle was then cut in half and as much of the gleba as possible was removed from the peridium using a sterilized spatula. Peridium and gleba tissues were lyophilized separately in 1.5 mL Eppendorf tubes, and the peridium was ground using a sterilized mortar and pestle. Using a sterilized stir bar and magnetic stirrer, 0.25 g of gleba and peridium were separately mixed into 25 mL of MM to produce 1% concentration solutions in separate 50 mL flasks and autoclaved for 15 min, resulting in two media conditions: *E. muricatus* peridium (EMP) and *E. muricatus* gleba (EMG). For the cuticle media (CUT), fifth instar larvae of *Otiorhyncus sulcatus* (Black Vine Weevil) were chosen because the closest insect pathogenic relative of *T. ophioglossoides*, which is *T. inflatum*, is a pathogen of beetle larvae. Larvae were dissected and decapitated using razors and sterilized spatulas were used to remove the organs and scrape as much of the hemolymph off of the cuticle as possible. Then, following the protocol from Andersen (1980), cuticles were soaked in 1% potassium tetraborate solution for 24 hr to remove any hemolymph or epidermal cells and dried completely at room temperature. After grinding with a mortar and pestle in liquid nitrogen, the insect cuticle was added to 25 mL of MM in 50 mL flasks at the same concentration as the *Elaphomyces* media, and autoclaved.

### Growth experiment

Strain CBS 100239 of *T. ophioglossoides* was grown on Corn Meal Agar for 1 wk to produce ample conidia. Plates were washed with molecular grade water, and filtered with Miracloth (EMD Millipore, Germany) to remove hyphae. A hemocytometer was used to estimate spore concentration, and 20uL of spore solution containing 3.6 × 10^4^ conidia was added to 2 mL of YM media in 3 mL wells in 12 multi-well (24 well) covered, individually-packaged disposable sterile plates (BD Falcon). After 7 d of growth, the plates were centrifuged for 1 min to pull hyphae and spore tissue to the bottoms of the wells, and then a micropipette with sterile filter tips was used to remove YM media from all the wells. Samples used for the YM growth condition were harvested at this time point. The tissue in the remaining wells was rinsed with molecular grade water, and then 2 mL of the alternative media was added to sets of replicate wells. Alternative media (EMP, EMG, and CUT) were then added in 2 mL aliquots to 3 replicate wells per condition (Supporting Information, Figure S2), resulting in 12 biological samples (4 treatments × 3 replicates). After 24 hr, tissue was harvested. To harvest tissue, plates were centrifuged for 30 sec and a micropipette was used to remove as much media as possible. *T. ophioglossoides* tissue was transferred via pipette to 1.5 mL Eppendorf tubes and frozen in liquid nitrogen and then immediately transferred to –80°, where samples were held until RNA extraction.

### Nucleic acid preparation and sequencing

RNA was extracted using the Qiagen RNeasy Plant kit following the manufacturer’s protocol. The Illumina TruSeq RNA Sample Preparation Kit v2 was used for RNA library construction, using manufacturer suggested protocols, including Agencourt AMPure magnetic beads for cleaning steps. The 12 RNA libraries described above were barcoded with the TruSeq kit adapters, multiplexed 12 deep per lane, and sequenced across 3 lanes as technical replicates (Auer and Doerge 2010), resulting in a total of 36 individual RNA-Seq datasets. Sequencing reactions were conducted for 51 single-end cycles on the Illumina HiSequation 2000 at the Center for Genome Research and Biocomputing at Oregon State University.

### Bioinformatic analyses

Sequenced reads were filtered based on Illumina quality flagging using the casava filtering programs from the Short Read Toolbox (Knaus 2014), and the first and last 5 bp were removed. This produced 45 bp trimmed and filtered RNA reads, which are available via the Sequence Read Archive at NCBI under the accession number SRP062415. The reads were aligned to the reference genome sequence of *T. ophioglossoides* (DDBJ/EMBL/Genbank accession LFRF00000000) using Bowtie 2 v. 2.0.6 with default settings (Langmead and Salzberg 2012). Unique reads were counted and sorted using a custom set of perl scripts. For each of the three biological replicates, there are three technical replicates. Verification of count variation among technical replicates was near Poisson distribution, suggesting it was safe to combine the counts from the technical replicates. For assessing differential expressed genes (DEG), the software package NBPSeq (Di *et al.* 2013) in R (Team 2013) was used to fit negative binomial regression models to RNA read counts where one of the regression coefficients corresponds to the log (base2) fold change between two treatments, for all pairwise comparisons. DEGs were assessed by testing whether that regression coefficient was 0 using a likelihood ratio test with higher-order asymptotic adjustment (Di 2015). The DEG test was performed on each gene separately and false discovery rates (q-values) were estimated according to Storey and Tibshirani (2003). The DEG analysis was repeated for each pair of the *T. ophioglossoides* replicates for EMP, EMG, YM, and CUT. A cutoff for DEGs was set at a false discovery rate q-value ≤ 0.10. Reads per thousand base pairs per million reads (RPKM) for each individual treatment was calculated using the following formula: [(total reads mapped to a gene across all replicates) / (the length of the gene (in bp) / 1000)] / (total reads for that treatment across all loci / 1,000,000).

To identify Interpro domains of *T. ophioglossoides* peptides, InterProScan v 5.44 (Jones *et al.* 2014) analysis was performed on the Discovery Environment of the iPlant Collaborative (https://de.iplantcollaborative.org/de/). The returned results were processed through customized scripts (provided by Cathy Gresham from Mississippi State University) to generate the Gene Ontology (GO) assignments. InParanoid 7 (Östlund *et al.* 2009) was run to identify homologs (orthologs and paralogs) between *T. ophioglossoides* and the references *Saccharomyces cerevisae*, *Neurospora crassa* and *Schizosaccharomyces pombe*. The resulting species pair homologs against the reference species (three of them, which have the most number of experimentally validated gene function assignments) were used to project gene homology based GO projections for *T. ophioglossoides*. The BiNGO 2.44 plugin (Maere *et al.* 2005) of Cytoscape 2.8 (Smoot *et al.* 2011) was used to identify statistically overrepresented GO categories of DEGs. Heatmap clustering in R (Team 2013) was based on default Euclidean distances as a part of the heatmap.2 program in the gplots package.

Hidden Markov models (HMMs) from dbCAN (Yin *et al.* 2012) were used to annotate CAZymes including GH18 family chitinases in the *T. ophioglossoides* and other hypocrealean genomes. PTH11-related GPCRs were identified using a custom HMM based on those PTH11-related GPCRs identified by Gao *et al.* (2011) in *M. anisopliae* and *M. acridum*, created for this study using the program Hmmer 3.0 (Eddy 2011). Prediction of the number of transmembrane helices associated with the PTH11-related proteins was performed by the TMHMM Server v. 2.0 (Krogh *et al.* 2001). The SignalP 4.1 webserver identified eukaryotic signal peptides with default cutoffs (Petersen *et al.* 2011). Additional annotations of DEGs were made using the published genome sequence annotations and further by BLAST searches against the NCBI nonredundant protein (nr) database with a minimum e-value of 1e-10. Secondary metabolite genes and clusters in *T. ophioglossoides* were based on those previously identified (Quandt *et al.* 2015a).

Phylogenetic analyses of protein families were performed by aligning sequences using MUSCLE v 3.8.31 (Edgar 2004) under default settings and manual removal of gaps, followed by maximum likelihood analysis using RAxML v 7.2.6 (Stamatakis 2006) and the Gamma model of rate heterogeneity, and the WAG substitution matrix with 100 bootstrap replicates.

### Data availability

Strain CBS100239 is available at CBS-KNAW Fungal Biodiversity Centre. File S1 contains read counts, log2 fold changes, and q-values for all DEGs for all pairwise comparisons, and individual RPKM values for each treatment for all genes. File S2 contains annotations of individual genes discussed in the paper along with individual RPKM values for each treatment. Raw sequence data are available in the NCBI Short Read Archive under the accession numbers SRR2179739, SRR2179742, SRR2179750, and SRR2179765. All *T. ophioglossoides* raw genome assembly and protein model, etc. files are available freely at https://github.com/alishaquandt.

## Results and Discussion

The growth conditions *E. muricatus* peridium media (EMP), *E. muricatus* gleba media (EMG), Yeast Malt media (YM), and insect cuticle media (CUT) resulted in 199 × 10^6^, 164 × 10^6^, 182 × 10^6^, and 214 × 10^6^ raw reads, respectively (Table S1). Using bowtie at default setting, 48.9% of these reads aligned to the genome of *T. ophioglossoides*, and 55.8% were unique alignments. Thus, there were 55.5 × 10^6^, 66.6 × 10^6^, 44.9 × 10^6^, and 113.9 × 10^6^ uniquely aligned reads for EMP, EMG, YM, and CUT, respectively. A large number of the reads aligned ambiguously (*i.e.*, they aligned at two or more locations within the genome), a result that is not surprising given the short read length of 45 bp. Still, despite this, there were more than a sufficient number of unambiguously aligned reads for downstream analyses. A larger percentage of uniquely aligned reads were obtained from fungi grown in cuticle media, possibly due to differences in RNA library qualities or quantifications prior to pooling. The differences in read counts between the conditions and replicates were accounted for in the statistical modeling used to identify DEGs.

Of the 10,134 *T. ophioglossoides* protein coding genes, 9659 (or 95.3%) were expressed under one or more of the growth conditions in this experiment (Quandt *et al.* 2015a). The most commonly expressed genes in all conditions were several heat shock proteins, a circadian clock-controlled protein, a DNA topoisomerase, translation elongation factor-1α, several genes involved in the citric acid cycle, and two proteins with no known function (Table 1).

**Table 1:** Most highly expressed *T. ophioglossoides* genes in *Elaphomyces* peridium media and corresponding ranked expression in other experimental conditions

Protein Model	EMP	EMG	YM	CUT	Annotation
TOPH_05155	1	1	1	1	HSP30
TOPH_08949*^a^*	2	2	2	4	No putative
TOPH_03606	3	4	3	7	CCG-6
TOPH_00884*^a^*	4	5	8	2	HSP70-2
TOPH_09013	5	3	4	6	SNF2-related protein
TOPH_08965	6	6	6	3	HSP30
TOPH_03227	7	7	13	5	HSP101
TOPH_03499	8	11	11	9	Globin-like protein
TOPH_02789	9	10	10	10	DNA topoisomerase 2
TOPH_09312*^a^*	10	8	5	12	No putative
TOPH_02300*^a^*	11	9	9	11	C-4 sterol methyl oxidase
TOPH_01093	12	12	7	13	Glyceraldehyde-3-phosphate dehydrogenase
TOPH_01292	13	14	14	15	Alcohol dehydrogenase I
TOPH_08416	14	17	35	14	Orphan
TOPH_08031	15	15	15	16	Endoplasmic oxidoreductin-1
TOPH_03470*^a^*	16	16	24	8	HSP90
TOPH_02137*^a^*	17	19	12	17	Pyruvate decarboxylase
TOPH_07279*^a^*	18	21	19	26	Ef-1α
TOPH_02581	19	18	17	22	Histone H3
TOPH_02697	20	20	18	20	No putative

EMP, *E. muricatus* peridium media; EMG, *E. muricatus* gleba media; YM, Yeast Malt media; CUT, *O. sulcatus* cuticle media; HSP, heat shock protein; Clock-controlled protein 6; SNF2, sucrose nonfermentation 2; Ef-1, elongation factor-1α.

a Statistically differentially expressed genes between two or more experimental conditions.

This experiment identified 5693 genes that were differentially expressed in at least one comparison of the growth conditions examined (File S1 and Table S2). The largest difference in gene expression was observed between EMG and CUT (47.6% of genes were differentially expressed), meaning that almost half of the transcriptome is differentially expressed between these two growth conditions. Similarly, 37% of genes were differentially expressed in EMP compared to CUT. These large differences in expression between the *Elaphomyces*-containing media and the insect cuticle media highlight the difference between the natural fungal host of *T. ophioglossoides* and the ancestral insect host of the genus *Tolypocladium*. The fewest number of DEGs (284, 3.1% of genes) were found between EMG and EMP, the two media containing *Elaphomyces* tissue. Expression changes between EMP, EMG, and CUT compared to YM, the rich media condition, were 8.6%, 16.3%, and 14.3% of genes, respectively. Of the 133 genes upregulated on EMP compared to both YM and CUT, only three were identified as orphans, having no homolog in the NCBI nr database or closely related taxa. One of these orphans (TOPH_00374) has a signal peptide and is only 99 amino acids in length (Quandt *et al.* 2015a), indicating that it may be a small secreted protein possibly involved in host–pathogen interactions, and it has moderate expression levels in EMP (RPKM = 12.3).

### Differential expression of PTH11-related G-protein coupled receptors

**Figure 1:**
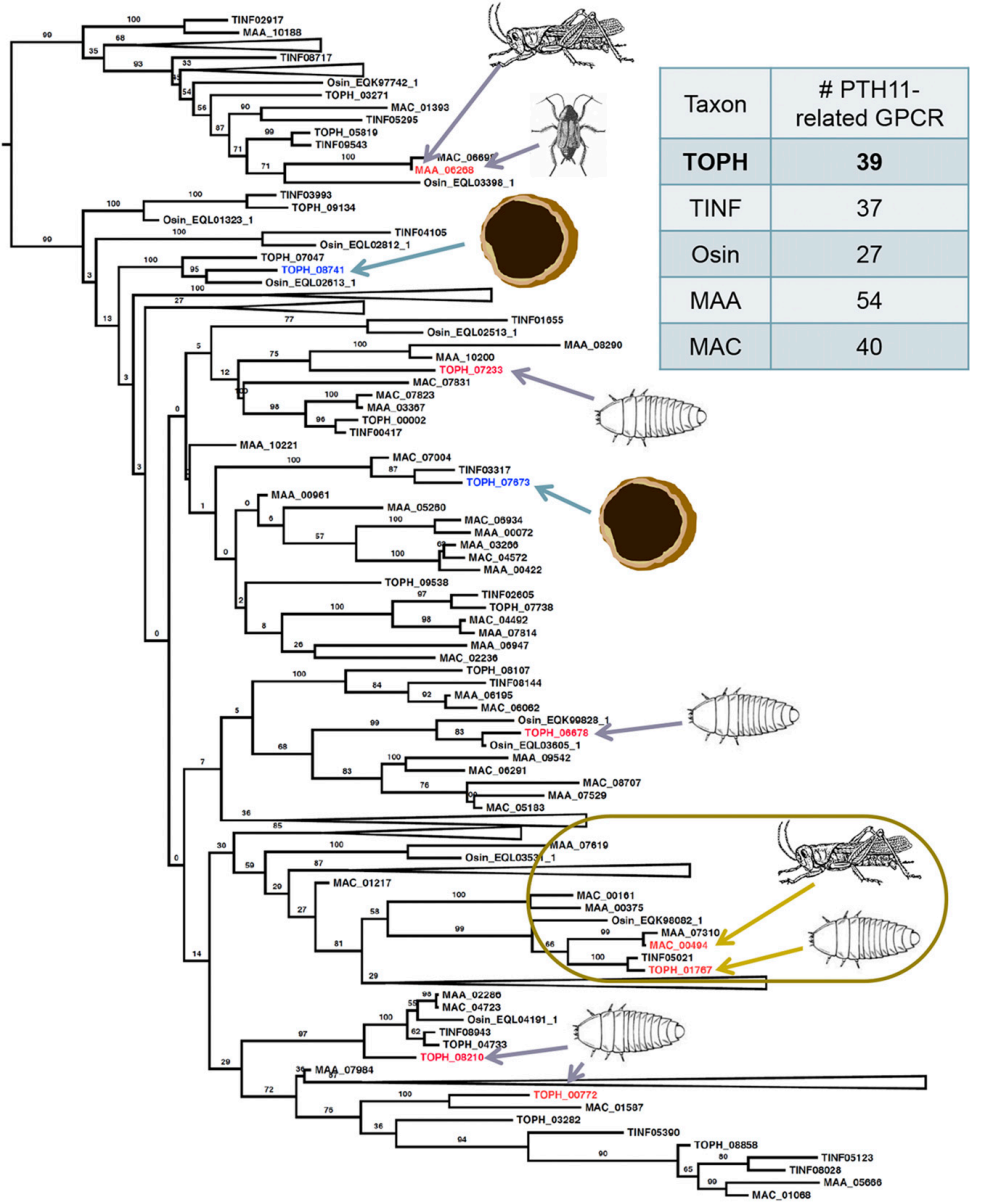
Phylogeny of PTH11-related GPCRs in *T. ophioglossoides* and closely related insect pathogens. PTH11-related GPCR phylogeny and numbers per taxon sampled. Genes that are upregulated in *T. ophioglossoides* on EMP are colored blue and marked with a truffle, and those that are upregulated on CUT are colored red and marked with a beetle larva. Genes that are upregulated in *Metarhizium* spp. on grasshopper or roach hosts are colored red and marked with the host(s) eliciting expression of those genes. The boxed area highlights the *T. ophioglossoides* homolog (TOPH_01767) of *M. acridum* (MAC_00494), which are both upregulated on insect cuticles. CUT, *O. sulcata* cuticle media; EMP, *E. muricatus* peridium media; GPCRs, G-protein-coupled receptors; Osin, *O. sinensis*; MAA, *M. robertsii*; MAC, *M. acidrum*; TINF, *T. inflatum*; TOPH, *T. ophioglossoides*.

Several PTH11-related GPCRs were identified in *T. ophioglossoides* and other related insect pathogens including *T. inflatum* and *Ophiocordyceps sinensis* of Ophiocordycipitaceae, and *M. robertsii* and *M. anisopliae* of the sister family Clavicipitaceae (Figure 1). Growth on EMP and CUT elicited different patterns of expression of these putative GPCRs in *T. ophioglossoides* (File S2). Specifically, TOPH_07673 and TOPH_08741 were upregulated on EMP compared to CUT. Of these, TOPH_08741 is part of a statistically well supported clade (Figure 1) containing only two other proteins; one from the moth pathogen *O. sinensis*, and the other a *T. ophioglossoides* paralog that is not differentially expressed between growth conditions. TOPH_07673 has one ortholog in *T. inflatum* and one in *M. acridum*, but not in *M. robertsii* or *O. sinensis*.

Several PTH11-related GPCRs were upregulated on CUT as compared to EMP, including TOPH_00772, TOPH_01767, TOPH_06678, TOPH_07233, and TOPH_08210. One of these, TOPH_01767, is orthologous to the PTH11-related GPCR, MAC_00494, of *M. acridum* that was upregulated on the cuticle of locust, its natural host (Figure 1). Encountering insect cuticle elicits the expression of the same receptor in both *M. acridum* and *T. ophioglossoides*, despite the loss of insect pathogenesis in the truffle parasite.

The combination of the large number of PTH11-related GPCRs and their differential expression supports the hypothesis that they function in the detection of different hosts of *Tolypocladium*. The upregulation of a *T. ophioglossoides* ortholog of a GPCR identified as highly expressed in a distantly related insect pathogen encountering insect tissue provides additional evidence for the PTH11-related GPCR host-recognition hypothesis examined in other studies (Gao *et al.* 2011).

### Chitinase expression

**Figure 2:**
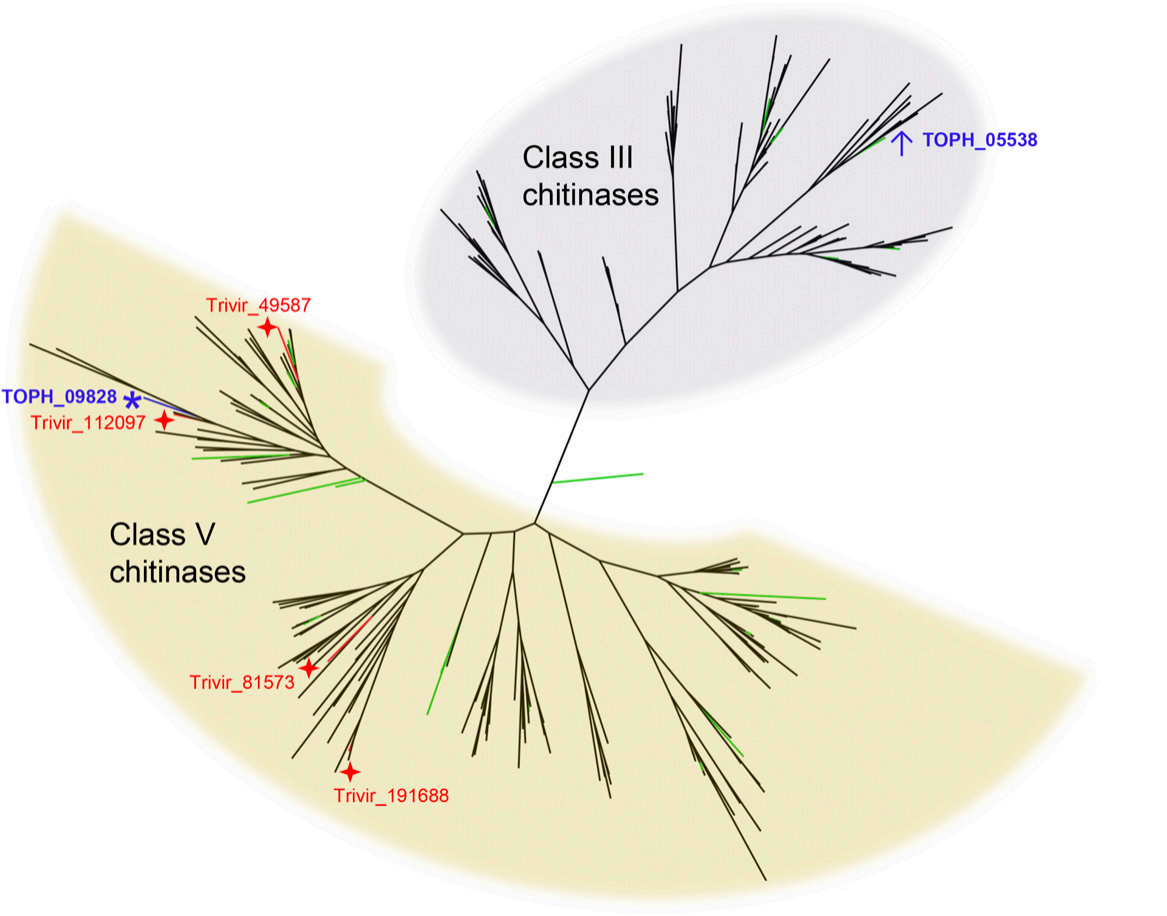
Phylogenetic relationships of expressed chitinases. Phylogeny of chitinases showing two major fungal classes. Green branches highlight those chitinases of *T. ophioglossoides*. The *T. ophioglossoides* chitinase (TOPH_09828) upregulated on EMP (*E. muricatus* peridium media, represented by an asterisk), which is closely related to one of the four *Tr. virens* chitinases upregulated during mycoparasitism (represented by stars), as identified by Gruber *et al.* (2011). The most highly expressed *T. ophioglossoides* chitinase (TOPH_05538) in all conditions (represented by a ↑) is part of the Class III chitinases, a phylogenetically distinct group. Taxa sampled: *Tr. virens*, *Tr. atroviride*, *Tr. reesei*, *Beauveria bassiana*, *Cordyceps militaris*, *Epichloë festucae*, *Claviceps purpurea*, *M. acridum*, *M. robertsii*, *O. sinensis*, *T. inflatum*, and *T. ophioglossoides*.

*T. ophioglossoides* possesses numerous GH18 chitinases in both Class III and Class V. One chitinase-like gene, TOPH_09828, was statistically upregulated during growth on both EMP and EMG compared to YM and CUT (Figure 2). This chitinase, while having undergone divergence at the sequence level and despite lacking carbohydrate binding modules (CBMs), is phylogenetically related to subgroup C of Class V chitinases. It possesses a signal peptide, which suggests that this chitinase is excreted into the extracellular environment (Figure S3), where it presumably degrades exogenous chitin. There is no homolog of this chitinase in the beetle pathogen *T. inflatum*, but TOPH_09828 is a homolog of one (Trive 112097) of the four subgroup C chitinases identified by Gruber *et al.* (2011) as upregulated in *Tr. virens* when grown during mycoparasitism and on fungal cell walls (Figure 2). It is not surprising that a *T. ophioglossoides* chitinase is upregulated on *Elaphomyces* tissue, as chitinases play a crucial role in breaking down fungal cell walls (Seidl 2008). The fact that this chitinase is closely related to those found to be upregulated during mycoparasitism by *Trichoderma* chitinases (Gruber *et al.* 2011) suggests two things. First, this chitinase may also play role in mycoparasitism (in Hypocreales mycoparasites), especially given the log (base2) fold change, 1.8 × and 2.0 × (*P* = 0.00002 and *P* = 0.0003) (File S1), between conditions and the moderately high RPKM, 36 and 37, on the media containing *Elaphomyces*, EMP, and EMG (respectively) compared to YM. Second, the shared ancestry of this particular gene suggests that the common ancestor of these two genera (and the four most divergent families to which they belong) may have had some mycoparasitic ability. This is intriguing because mycoparasites are found in all of the most divergent families (Kepler *et al.* 2012), and previous studies using a large phylogenetic sampling found equivocal support for the ancestral character state of the ancestor of these families as either pathogens of insect, fungi, or plants (Spatafora *et al.* 2007; Sung *et al.* 2008).

The most highly expressed chitinase under all conditions was TOPH_05538, a Class III chitinase that does not possess any CBMs or a signal peptide (Figure 2). It was the 33rd most common transcript in EMP, 30th in EMG, 35th in YM, and 56th in CUT. Statistically, TOPH_05538 was upregulated in EMP and EMG compared to CUT, but not to YM. The lack of a signal peptide suggests that this highly expressed chitinase may play an endogenous role within *T. ophioglossoides*.

Other CAZymes upregulated in EMP and EMG compared to YM and CUT include two β-1,3-glucanases, which break bonds in a common component of cell walls of filamentous fungi, β-1,3-glucans (polymers of glucose). Both of these glucanases have signal peptides indicating that they are secreted proteins, but one (TOPH_03534) is annotated as part of the CAZy family GH64, which are endo-acting and implicated in fungal cell wall degradation, whereas the other (TOPH_03859) is exo-acting and possesses an extra chitinase-like domain. β-1,3-glucans are one of the most abundant carbohydrates in the cell walls of *Aspergillus fumigatus* (Latgé *et al.* 2005; Latgé 2007, 2010), a close relative of *Elaphomyces*, the host of *T. ophioglossoides*, and expression of these β-1,3-glucanases is hypothesized here to facilitate *T. ophioglossoides* parasitism of its host fungus.

### Expression of adhesins

**Figure 3:**
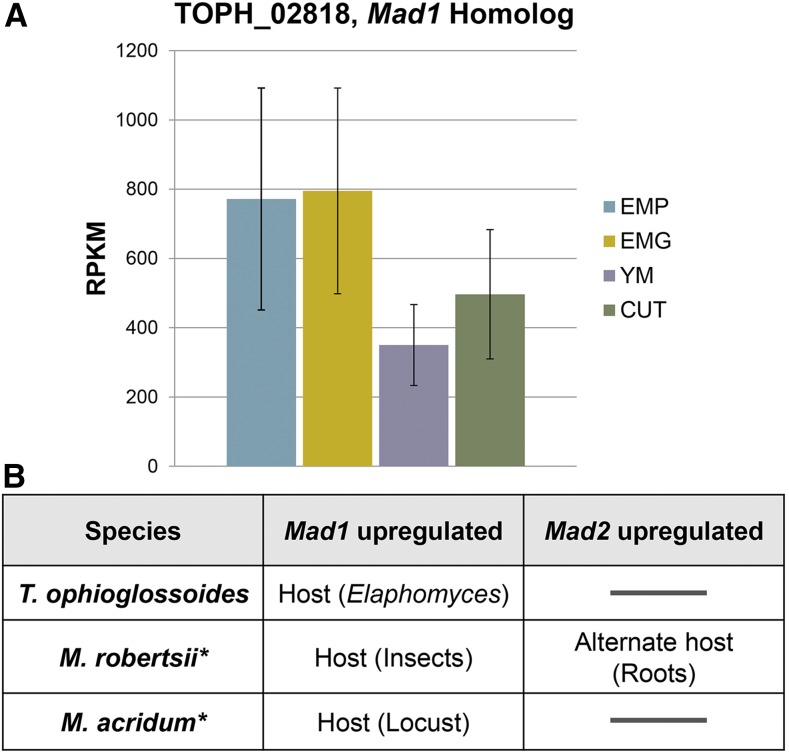
*Mad1* expression. (A) RPKM and corresponding 95% confidence intervals of *Mad1* homolog in *T. ophioglossoides* during growth in four experimental conditions. (B) Upregulation of *Mad1* and *Mad2* homologs in three hypocrealean spp. from data collected in this study combined with data from *M. robertsii* and *M. acridum* in other studies (Wang and Leger 2005; Wang *et al.* 2005; Gao *et al.* 2011). CUT, *O. sulcata* cuticle medium; EMG, *E. muricatus* gleba media; EMP, *E. muricatus* peridium media; RPKM, reads per thousand base pairs per million reads; YM, Yeast Malt medium.

One of the most highly expressed *T. ophioglossoides* genes in EMP was the ortholog (TOPH_02818) of the *M. robertsii* adhesin gene, *Mad1*. Statistically, the *Mad1* homolog is upregulated on both EMP and EMG compared to YM (File S1), and somewhat upregulated in EMG compared to CUT (log2 fold change = 0.679, *P* = 0.07, q = 0.07), although the 95% confidence intervals overlap to some extent (Figure 3). MAD1 was identified in *M. robertsii* because of high expression during insect pathogenesis (Wang *et al.* 2005), and was later found to be involved in both cytoskeletal orientation and blastospore formation (Wang and St Leger 2007). Blastospores are a yeast-like growth morphology frequently taken on by fungi when growing in liquids, where filamentous growth can be restricted, and it is unlikely that *T. ophioglossoides* produces blastospores during its infection of *Elaphomyces* truffles in nature. While this experiment was conducted exclusively using liquid media (where blastospore growth could have been induced but was not monitored during the experiment), the *Mad1* homolog was still statistically upregulated on *Elaphomyces* media (EMP and EMG) compared to the growth on YM. This indicates that MAD1 may play a role in host infection that is not solely involved in changes in growth morphology. The upregulation of *Mad1* on EMP and EMG could be related to adhesion to *Elaphomyces* cells. It is also possible that the *T. ophioglossoides* MAD1 homolog plays a role in increased overall growth on its natural host and progression of the cell cycle. This is supported by the fact that all studies examining *Mad1* expression, including this one, have found it to be highly expressed regardless of condition (Figure 3) (Wang *et al.* 2005; Wang and St Leger 2007). Furthermore, if MAD1 is necessary for normal cellular functioning this could explain why its deletion in *M. robertsii* caused a reduction in insecticidal ability.

In contrast, the *T. ophioglossoides* ortholog of *Mad2* (TOPH_07267) had low expression under all of the growth conditions tested and was not differentially expressed (File S2). This protein has been proposed to promote attachment to the plant surface (Wang and St Leger 2007), and while this condition was not tested in our experiment, *T. ophioglossoides* is not known to grow in association with plants.

### Overrepresented gene ontologies

**Figure 4:**
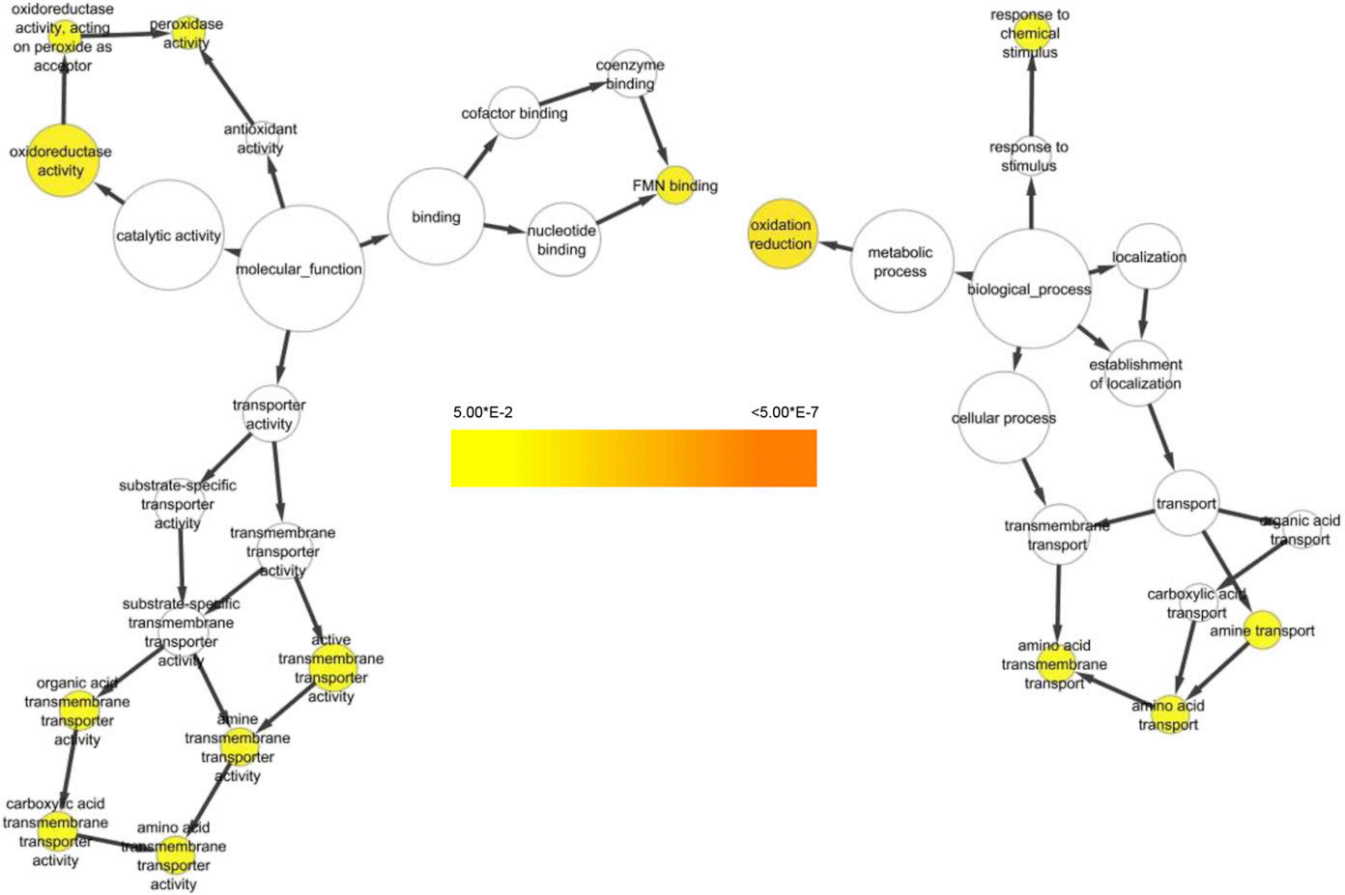
Upregulated Gene Ontology (GO) categories in EMP (*E. muricatus* peridium media). BiNGO network of statistically overrepresented GO categories in the 360 *T. ophioglossoides* genes upregulated in EMP compared to YM (Yeast Malt medium). Colors are based on p-values and follow the scale provided.

BiNGO analysis of the Gene Ontology (GO) categories assigned to *T. ophioglossoides* DEGs identified three major categories of GOs that were statistically overrepresented in EMP compared to YM (Figure 4). Two GO terms, *GO:0055114* and *GO:0016491*, corresponding to the ontologies oxidation reduction and oxidoreductase, respectively, are part of a large group of redox-related GOs overrepresented in *T. ophioglossoides* genes upregulated in EMP (Figure 4 and Table S3). This result suggests that certain types of oxidative stress may be employed by *T. ophioglossoides* when it encounters its host and could play a role in its mycoparasitic ability.

The second most overrepresented GO during *T. ophioglossoides* growth on EMP is FMN binding (*GO:0010181*) (Table S3). Flavin mononucleotide (FMN) is the coenzyme of flavoprotein oxidoreductase enzymes, and this GO category is a child of many parent GOs, including cofactor binding, signal transduction activity, receptor activity, and ion binding. It is possible that this category of proteins is involved in redox reactions as well, and one of the proteins in this category is annotated as cytochrome p450 (TOPH_05009). Upregulation of enzymes involved in oxidation and reduction has been observed in studies examining fungi growing on, or near, their hosts (DiGuistini *et al.* 2007). Some studies have examined oxidoreductases used by plant-pathogenic fungi for detoxification of the host environment (Idnurm and Howlett 2001). Also, a high number of both cytochrome p450s and monoxygenases were expressed in *Metarhizium* spp. grown on host tissue (Gao *et al.* 2011).

The other major group of overrepresented GO categories that were upregulated in EMP is related to transmembrane transport (Figure 4). Specifically, transmembrane transporters of amino acids, organic acids, and carboxylic acids were overrepresented. The transport of amino acids and their derivatives is known to be important for cellular processes including energy generation, cell wall synthesis, and intercellular signaling (Saier 2000), indicating this may be due to an increase in *T. ophioglossoides* growth and proliferation in the presence of *Elaphomyces*. Overall growth of tissue was not quantified at harvesting, but based on visual inspection of the plates, the wells containing EMP had the most tissue at the end of the experiment.

### Expression patterns of secondary metabolite genes

**Figure 5:**
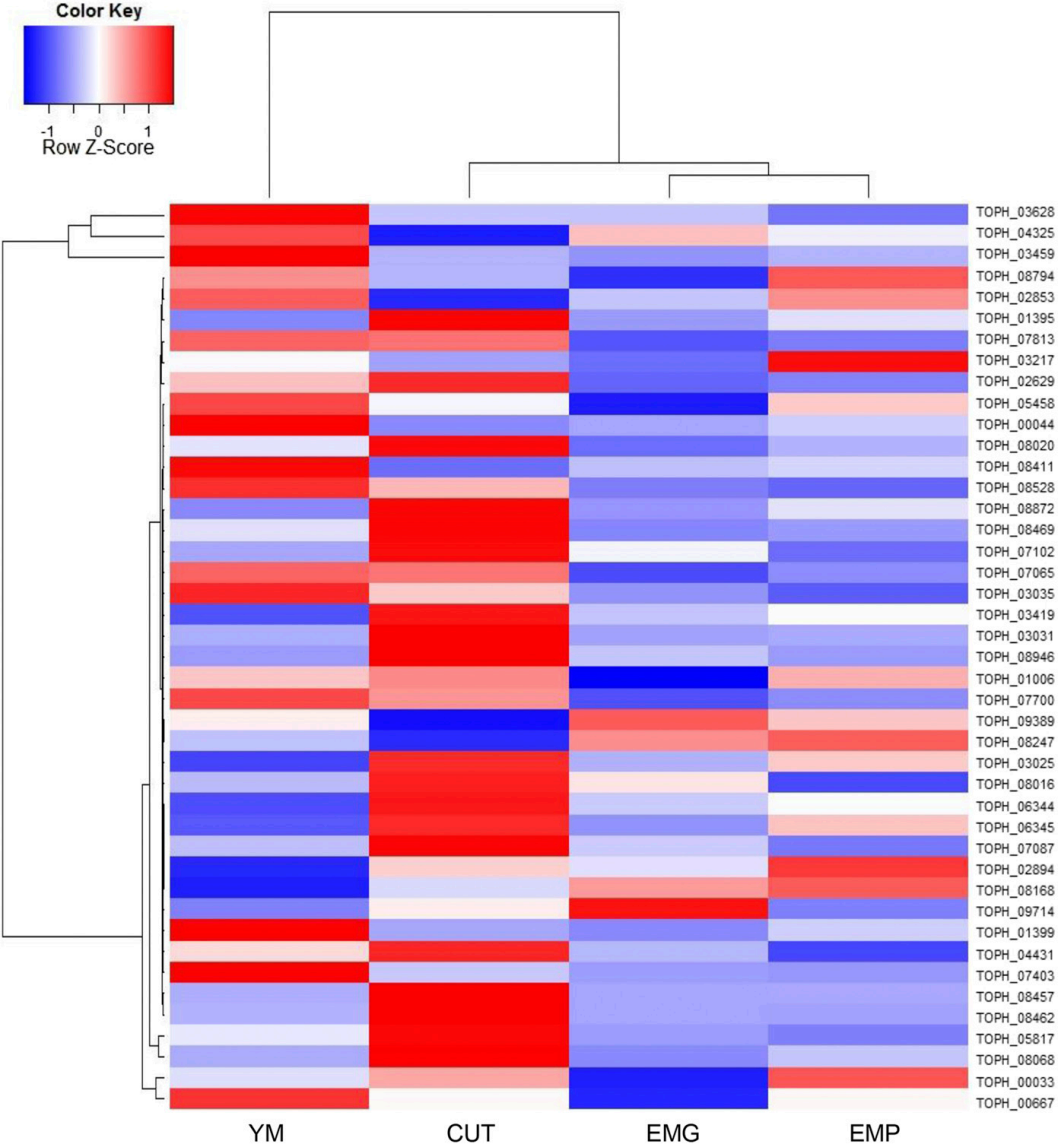
Secondary metabolite expression. Heatmap of RPKM of secondary metabolite core genes (NRPS, PKS, Terpene Synthases) in *T. ophioglossoides* under the experimental conditions. Row scaled Z-scores are based on normalized standard deviation units. CUT, *O. sulcata* cuticle media; EMG, *E. muricatus* gleba medium; EMP, *E. muricatus* peridium medium; NRPS, nonribosomal peptide synthetases; PKS, polyketide synthases; RPKM, reads per thousand base pairs per million reads; YM, Yeast Malt medium.

Based on DEGs and the RPKMs for each of the core secondary metabolite (SM) genes, including nonribosomal peptide synthetases (NRPSs) and polyketide synthases (PKSs), secondary metabolism appears to be reduced when *T. ophioglossoides* is grown on tissue containing its host (Figure 5, Figure S1, and File S2). The most highly expressed SM core genes were an NRPS-like gene (TOPH_03459) and two terpene synthases (TOPH_03628 and TOPH_04325). The NRPS-like SM gene, TOPH_03459, is statistically upregulated in YM compared to the other conditions (File S2). There are no known products for these gene clusters.

Some of the secondary metabolite core genes are upregulated exclusively during growth on insect cuticle, while others are equally expressed in YM and CUT. Notable clusters that are upregulated on cuticle include almost the entire peptaibiotic cluster around the NRPS gene (TOPH_08469) (Quandt *et al.* 2015a), including the two PKS genes (TOPH_08462 and TOPH_08457) in that cluster (Figure S1 and File S2). Other SM core genes that are upregulated in CUT compared to EMP and EMG include a terpene synthase (TOPH_08068), an NRPS (TOPH_05817), a PKS (TOPH_01395), and a putative extracellular siderophore synthetase NRPS (TOPH_02629) (File S2). The upregulation of SM genes when *T. ophioglossoides* is grown on insect cuticle could be a response to a stressful or potentially foreign environment, or could represent an ancestral response to the insect environment. Secondary metabolism has not been a topic of discussion in studies examining genes expressed on insect tissue in *M. anisopliae* (Wang and St Leger 2005; Wang *et al.* 2005), but several studies have reported secondary metabolite production during infection of insect hosts in that species (Roberts 1981; Wang *et al.* 2012). The low expression of *T. ophioglossoides* secondary metabolite core genes during growth on *Elaphomyces* media mirrors reports from mycoparasitism assays in *Tr. atroviride*. The cause of this differential expression remains to be determined, but *T. ophioglossoides* must survive in soil, where it interacts with a large number of microorganisms including bacteria, and secondary metabolism could be involved in defense against these. There are also many bacteria living within the fruiting body of *Elaphomyces* (Quandt *et al.* 2015b), and it could be that during growth on its host in nature, *T. ophioglossoides* may express higher amounts of secondary metabolites, but this was not addressed within the context of this study.

Simulating growth under different host conditions provides insights into how pathogenic fungi perceive and begin to antagonize host tissue, and how fungi may regulate similar gene repertoires to achieve host jumping. Almost half of the genes in the *T. ophioglossoides* genome were differentially expressed depending on whether the growth media contained its host, the truffle *Elaphomyces*, or an unnatural host, the beetle, *Otiorhyncus*. This experiment provides the first glimpse into mycoparasitic interactions in a non-*Trichoderma* system, and provides some hypotheses for how a gene repertoire very similar to close insect pathogenic relatives may be differentially expressed to enable parasitism of other fungi. Some patterns of *T. ophioglossoides* expression mirror those seen in *Trichoderma* mycoparasitism assays, including an upregulation of a divergent homolog of subgroup C of Class V chitinase and a downregulation of secondary metabolism. PTH11-related GPCRs were found to be differentially expressed under the different growth conditions, and two GPCRs were identified as candidate receptors involved in *Elaphomyces* recognition. Analysis of GO categories that were overrepresented when *T. ophioglossoides* was grown on its host, revealed that redox reactions and transmembrane transport of amino acids and their derivatives were dominant. The adhesin gene, *Mad1*, was highly expressed during all growth conditions, but was significantly upregulated in media containing *Elaphomyces* peridium and gleba as compared to the rich media. *Mad2* showed low expression under all conditions. Some of these transcriptional responses in *T. ophioglossoides* may represent expression of plesiomorphic responses to ancestral hosts (*i.e.*, insects) but many appear to be specific to the current host, *Elaphomyces*. Future studies could examine both the insect pathogenic *Tolypocladium* species’ responses to similar media conditions as a comparison to those expressed in this study, or develop more traditional genetic techniques (*e.g.*, gene knockouts) or quantitative measures (*e.g.*, qPCR) to verify the results identified here.

## Supplementary Material

Supporting Information
