# Supplementary material for: Differential Expression of Genes Involved in Host Recognition, Attachment, and Degradation in the Mycoparasite Tolypocladium ophioglossoides
Source: G3 (Bethesda). 2016 Jan 20;6(3):731–41. doi: 10.1534/g3.116.027045 (PMC4777134; doi:10.1534/g3.116.027045)
Supplement: Supporting Information [file supp_6_3_731__index.html]

Differential Expression of Genes Involved in Host Recognition, Attachment, and Degradation in the Mycoparasite Tolypocladium ophioglossoides — Supporting Information 

# Differential Expression of Genes Involved in Host Recognition, Attachment, and Degradation in the Mycoparasite *Tolypocladium ophioglossoides*

## Supporting Information for Quandt *et al.*, 2016

**Files in this Data Supplement:**

- Table S1 - Reads obtained in the experiment for each biological and technical replicate in each growth condition. (.pdf, 129 KB)
- Table S2 - Numbers of differentially expressed genes identified. (.pdf, 125 KB)
- Table S3 - Overrepresented GO terms during growth on truffle peridium compared to rich media. (.pdf, 139 KB)
- File S1 - Spreadsheet with raw reads, log2 fold change values, q-values, and p-values for all differentially expressed genes for each of the pairwise treatment comparisons, and the individual treatment RPKM values for all expressed genes. (.xlsx, 3,061 KB)
- File S2 - RPKM values for all chitinases, PTH11-related GPCRs, secondary metabolite core genes, the TOPH\_08469 peptaibiotic gene cluster, and *Mad1* and *Mad2*. Also indicated is whether or not these genes were statistically differentially expressed in one or more treatment comparisons. (.xlsx, 24 KB)
- Figure S1 - Heatmap of peptaibiotic TOPH\_08469 cluster expression. RPKM expression values of *T. ophioglossoides* during the four different growth conditions, Yeast Malt (YM), minimal media containing *Elaphomyces muricatus* peridium (EMP), minimal media containing insect cuticle (CUT), and minimal media containing *E. muricatus* gleba (EMG). (.tif, 19,801 KB)
- Figure S2 - Experimental design for the study. Biological replication of the growth conditions and technical replication across three lanes of Illumina sequencing are shown. Abbreviations for the growth conditions as in Figure S1. (.tif, 8.816 KB)
- Figure S3 - Schematic of the chitinase-like *T. ophioglossoides* protein model TOPH\_09828. Numbers below functional domains/ regions correspond to amino acids, and the amino acid sequence is provided below. (.tif, 8,815 KB)
