# Supplementary material for: Differential Expression of Genes Involved in Host Recognition, Attachment, and Degradation in the Mycoparasite Tolypocladium ophioglossoides
Source: G3 (Bethesda). 2016 Jan 20;6(3):731–41. doi: 10.1534/g3.116.027045 (PMC4777134; doi:10.1534/g3.116.027045)
Supplement: Supporting Information [file supp_g3.116.027045_TableS1.pdf]

Table S1. Reads obtained in the experiment for each biological and technical replicate in each growth condition. Number of reads obtained per biological and technical replicate after passing the Illumina quality filter. Technical replicates of the same biological replicate are shown in the same color, and replicates of the same treatment are shown in different hues of the same color.

YM	Read Count	EMP	Read Count	CUT	Read Count	EMG	Read Count
YM1 lane5	16984502	EMP5 lane5	19455818	CUT4 lane5	21557554	EMG3 lane 1	17774680
YM1 lane6	16884615	EMP5 lane6	19493806	CUT4 lane6	21607741	EMG3 lane6	17510987
YM1 lane7	16746146	EMP5 lane7	19391547	CUT4 lane7	21475196	EMG3 lane7	17505495
YM3 lane5	23053582	EMP6 lane5	19876712	CUT6 lane5	25456069	EMG6 lane1	18222718
YM3 lane6	23099212	EMP6 lane6	19925044	CUT6 lane6	25496300	EMG6 lane6	17990853
YM3 lane7	22977797	EMP6 lane7	19803261	CUT6 lane7	25379915	EMG6 lane7	17937229
YM4 lane5	20631881	EMP8 lane5	20342464	CUT8 lane5	24398390	EMG9 lane1	18984453
YM4 lane6	20643393	EMP8 lane6	20274380	CUT8 lane6	24471158	EMG9 lane6	18824350
YM4 lane7	20500989	EMP8 lane7	20238837	CUT8 lane7	24357937	EMG9 lane7	18824350
Total	181522117	Total	199040706	Total	214200260	Total	163575115
