# Supplementary material for: Differential Expression of Genes Involved in Host Recognition, Attachment, and Degradation in the Mycoparasite Tolypocladium ophioglossoides
Source: G3 (Bethesda). 2016 Jan 20;6(3):731–41. doi: 10.1534/g3.116.027045 (PMC4777134; doi:10.1534/g3.116.027045)
Supplement: Supporting Information [file supp_g3.116.027045_TableS2.pdf]

Table S2. Numbers of differentially expressed genes identified. Numbers of *T. ophioglossoides* statistically significant differentially expressed genes for each pairwise treatment comparisons (q-value < 0.1).

Comparison	# of DEGs
**EMG and CUT**	4331
**EMP and CUT**	3363
**EMG and YM**	1486
**YM and CUT**	1303
**EMP and YM**	786
**EMP and EMG**	284
