# Supplementary material for: Differential Expression of Genes Involved in Host Recognition, Attachment, and Degradation in the Mycoparasite Tolypocladium ophioglossoides
Source: G3 (Bethesda). 2016 Jan 20;6(3):731–41. doi: 10.1534/g3.116.027045 (PMC4777134; doi:10.1534/g3.116.027045)
Supplement: Supporting Information [file supp_g3.116.027045_TableS3.pdf]

Table S3. Overrepresented GO terms during growth on truffle peridium compared to rich media. Statistically overrepresented GO terms identified using BiNGO from *T. ophioglossoides* genes upregulated on EMP compared to YM, and the corresponding gene models in each category.

GO-ID	Description	p-val	correct p-val	cluster freq	total freq	Protein models
55114	**oxidation reduction**	4.91E-06	3.70E-03	42/205 20.4%	524/5167 10.1%	TOPH_05009 TOPH_08668 TOPH_05655 TOPH_06301 TOPH_01520 TOPH_06771 TOPH_01519 TOPH_02834 TOPH_02373 TOPH_05757 TOPH_01110 TOPH_02507 TOPH_08259 TOPH_06457 TOPH_01967 TOPH_02434 TOPH_03747 TOPH_01965 TOPH_00010 TOPH_09943 TOPH_07165 TOPH_01108 TOPH_04762 TOPH_00730 TOPH_07628 TOPH_07487 TOPH_03670 TOPH_05310 TOPH_03281 TOPH_06636 TOPH_04789 TOPH_06021 TOPH_06186 TOPH_09004 TOPH_05056 TOPH_06565 TOPH_08404 TOPH_04264 TOPH_04245 TOPH_05576 TOPH_05267 TOPH_07739
10181	**FMN binding**	2.72E-05	1.02E-02	7/205 3.4%	24/5167 0.4%	TOPH_07165 TOPH_05009 TOPH_03747 TOPH_00730 TOPH_06771 TOPH_02834 TOPH_02373
16491	**Oxidoreductase**	4.44E-05	1.12E-02	47/205 22.9%	670/5167 12.9%	TOPH_07679 TOPH_07377 TOPH_05009 TOPH_08668 TOPH_05655 TOPH_06301 TOPH_01520 TOPH_06771 TOPH_01519 TOPH_02834 TOPH_02373 TOPH_05757 TOPH_01110 TOPH_02507 TOPH_07609 TOPH_08259 TOPH_06457TOPH_01967 TOPH_03747 TOPH_02434 TOPH_01965 TOPH_00010 TOPH_06725 TOPH_07165 TOPH_09943 TOPH_01108 TOPH_04762 TOPH_00730 TOPH_07628 TOPH_07487 TOPH_03670 TOPH_05310 TOPH_03281 TOPH_06636 TOPH_04789 TOPH_06021 TOPH_03498 TOPH_06186 TOPH_09004 TOPH_05056 TOPH_06565 TOPH_08404 TOPH_04264 TOPH_04245 TOPH_05576 TOPH_05267 TOPH_07739
46943	**carboxylic acid transmembrane transporter activity**	3.25E-04	4.22E-02	8/205 3.9%	45/5167 0.8%	TOPH_07839 TOPH_07915 TOPH_01966 TOPH_02728 TOPH_01447 TOPH_07883 TOPH_01331 TOPH_05834
5342	**organic acid transmembrane transporter activity**	3.25E-04	4.22E-02	8/205 3.9%	45/5167 0.8%	TOPH_07839 TOPH_07915 TOPH_01966 TOPH_02728 TOPH_01447 TOPH_07883 TOPH_01331 TOPH_05834
4601	**peroxidase activity**	4.19E-04	4.22E-02	4/205 1.9%	10/5167 0.1%	TOPH_06021 TOPH_05056 TOPH_08668 TOPH_01520
16684	**oxidoreductase activity, acting on peroxide as acceptor**	4.19E-04	4.22E-02	4/205 1.9%	10/5167 0.1%	TOPH_06021 TOPH_05056 TOPH_08668 TOPH_01520
22804	**active transmembrane transporter activity**	5.21E-04	4.22E-02	15/205 7.3%	144/5167 2.7%	TOPH_07826 TOPH_07915 TOPH_07839 TOPH_02728 TOPH_04759 TOPH_01447 TOPH_08787 TOPH_05834 TOPH_05431 TOPH_01966 TOPH_02276 TOPH_02729 TOPH_07883 TOPH_01331 TOPH_06819

15171	**amino acid transmembrane transporter activity**	7.28E-04	4.22E-02	7/205 3.4%	39/5167 0.7%	TOPH_07839 TOPH_07915 TOPH_01966 TOPH_02728 TOPH_07883 TOPH_01331 TOPH_05834
3333	**amino acid transmembrane transport**	7.28E-04	4.22E-02	7/205 3.4%	39/5167 0.7%	TOPH_07839 TOPH_07915 TOPH_01966 TOPH_02728 TOPH_07883 TOPH_01331 TOPH_05834
5275	**amine transmembrane transporter activity**	7.28E-04	4.22E-02	7/205 3.4%	39/5167 0.7%	TOPH_07839 TOPH_07915 TOPH_01966 TOPH_02728 TOPH_07883 TOPH_01331 TOPH_05834
15837	**amine transport**	7.28E-04	4.22E-02	7/205 3.4%	39/5167 0.7%	TOPH_07839 TOPH_07915 TOPH_01966 TOPH_02728 TOPH_07883 TOPH_01331 TOPH_05834
6865	**amino acid transport**	7.28E-04	4.22E-02	7/205 3.4%	39/5167 0.7%	TOPH_07839 TOPH_07915 TOPH_01966 TOPH_02728 TOPH_07883 TOPH_01331 TOPH_05834
42221	**response to chemical stimulus**	8.01E-04	4.31E-02	6/205 2.9%	29/5167 0.5%	TOPH_06021 TOPH_05056 TOPH_08668 TOPH_02276 TOPH_08912 TOPH_01520
