# Supplementary figures and images for: Differential Expression of Genes Involved in Host Recognition, Attachment, and Degradation in the Mycoparasite Tolypocladium ophioglossoides
Source: G3 (Bethesda). 2016 Jan 20;6(3):731–41. doi: 10.1534/g3.116.027045 (PMC4777134; doi:10.1534/g3.116.027045)

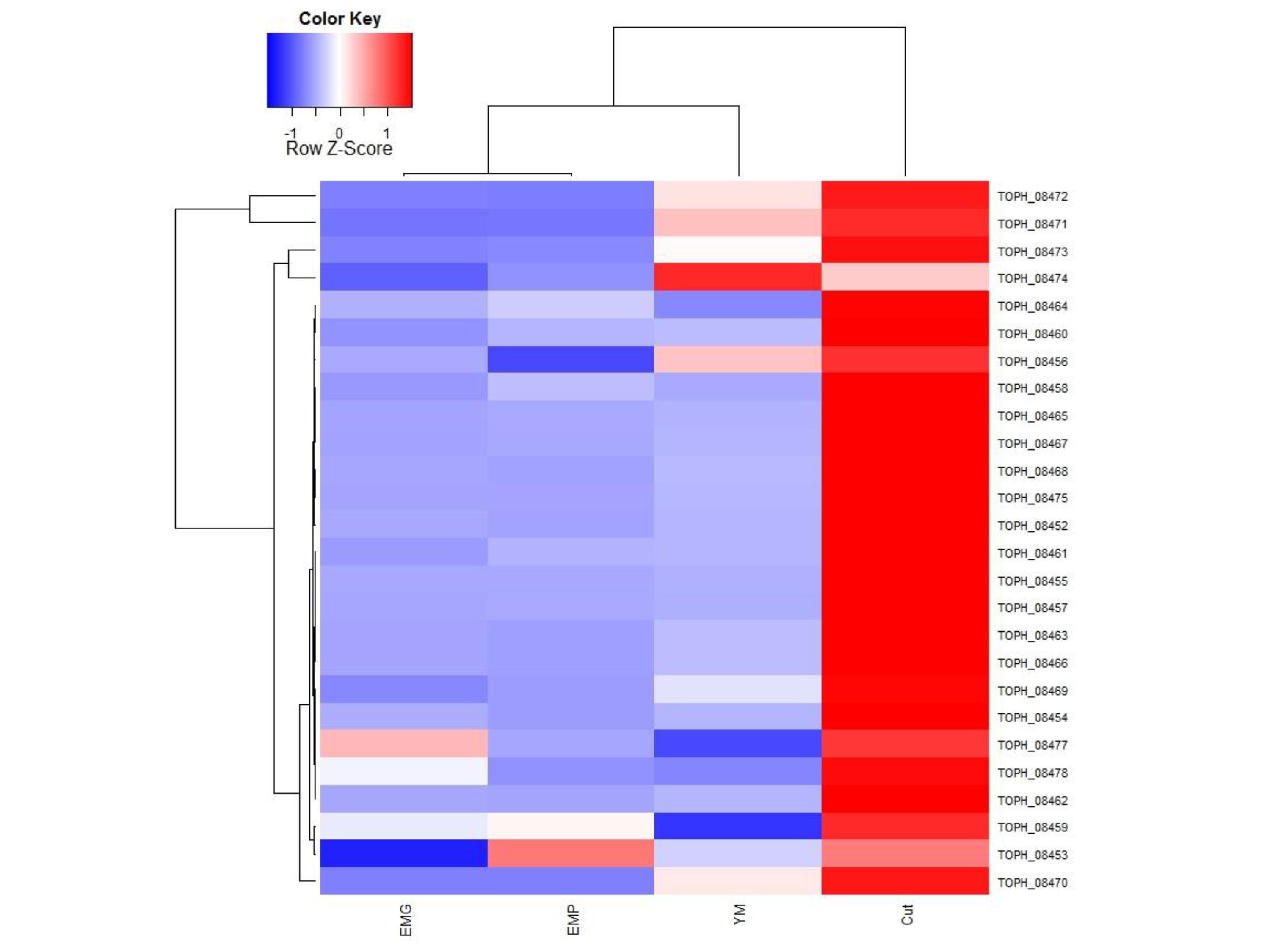

Supplement: Supporting Information [file supp_g3.116.027045_FigureS1.tif]

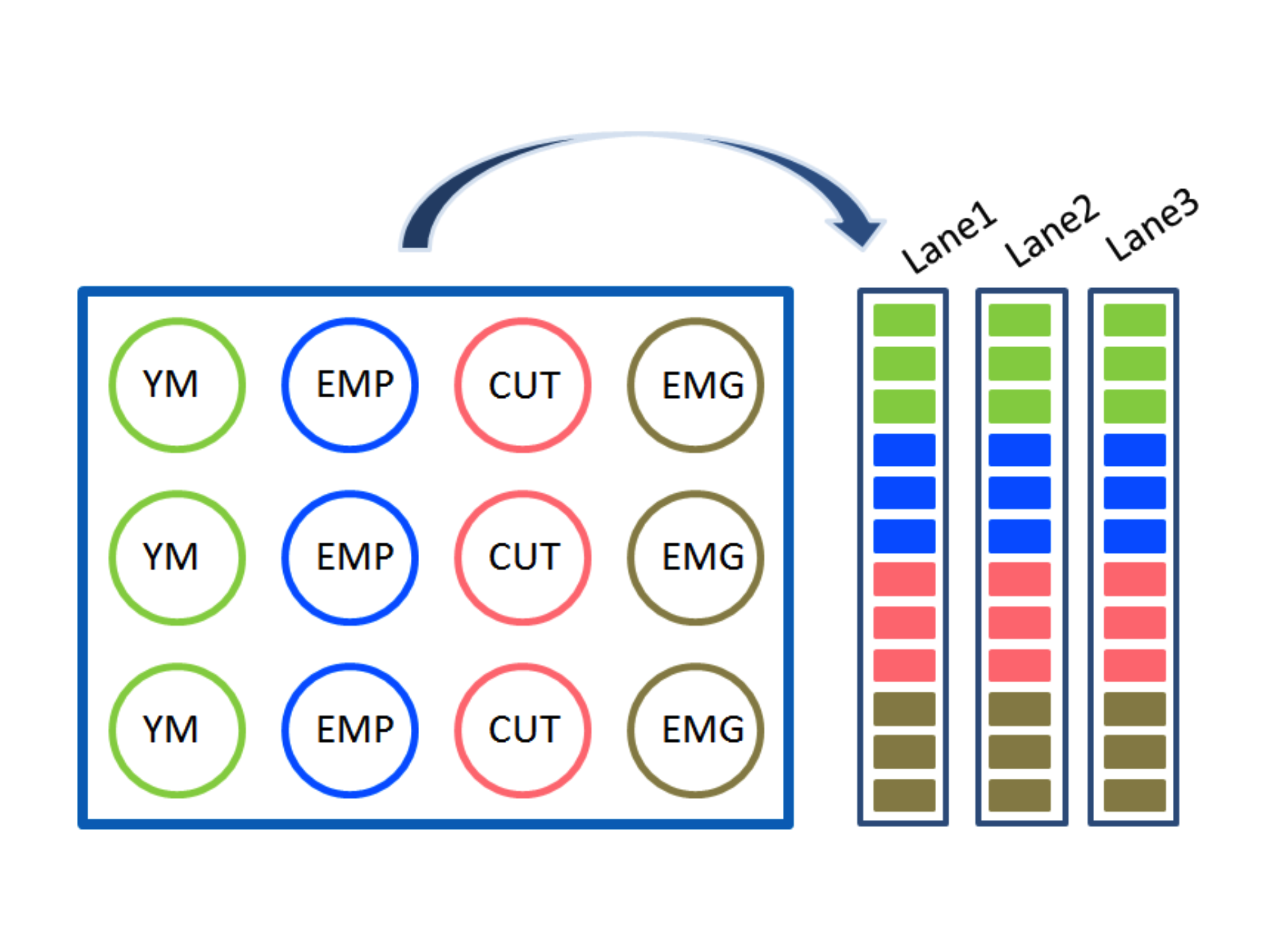

Supplement: Supporting Information [file supp_g3.116.027045_FigureS2.tif]

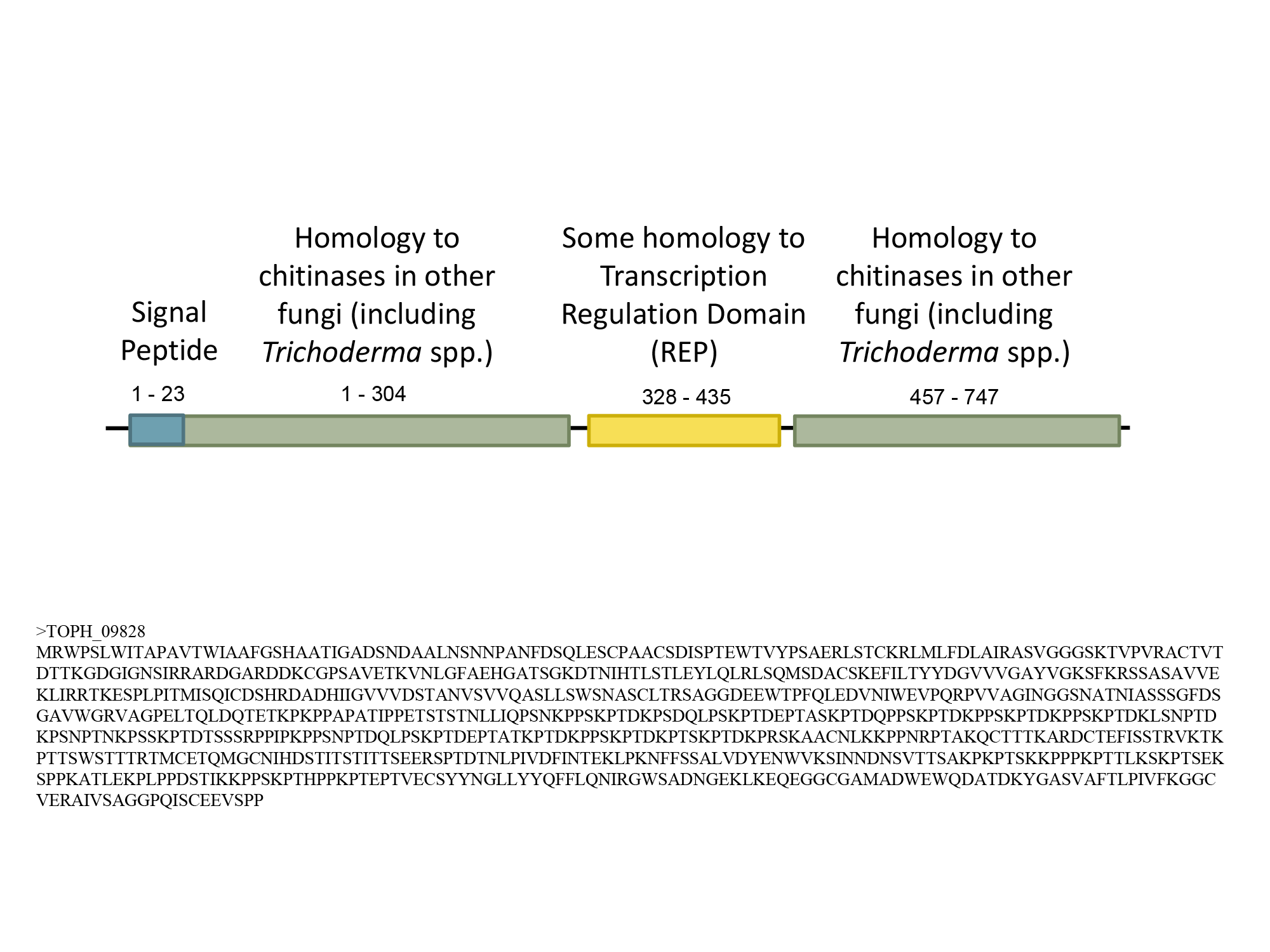

Supplement: Supporting Information [file supp_g3.116.027045_FigureS3.tif]
